# Clinical and Individual Factors Associated with Smoking Quit Attempts among Adults with COPD: Do Factors Vary with Regard to Race?

**DOI:** 10.3390/ijerph110403717

**Published:** 2014-04-03

**Authors:** Dana S. Mowls, Vinay K. Cheruvu, Melissa D. Zullo

**Affiliations:** 1 Department of Epidemiology and Biostatistics, College of Public Health, Kent State University, Kent, OH 44242, USA; E-Mails: dmowls@ouhsc.edu (D.S.M.); vcheruvu@kent.edu (V.K.C.); 2 Department of Biostatistics & Epidemiology, College of Public Health, University of Oklahoma Health Sciences Center, Oklahoma City, OK 73103, USA

**Keywords:** chronic obstructive pulmonary disease, COPD, smoking, cessation, quit attempts, tobacco, respiratory, race

## Abstract

Only half of adults with chronic obstructive pulmonary disease (COPD) report a smoking quit attempt in the past year. Adults with COPD have frequent encounters with the healthcare system that are opportunities for health behavior interventions that support quit attempts. The purpose of this research was to examine individual- and clinical-level factors associated with smoking quit attempts in adults with COPD. Cross-sectional data were from the 2011 Behavioral Risk Factor Surveillance System. Race-stratified, weighted logistic regression examined factors associated with quit attempt among current smokers with COPD. Overall, quit attempt was reported by 65% (95% confidence interval (CI): 61.9, 67.5) of adults and was more likely among blacks than whites (*p* < 0.0001). Among whites with COPD quit attempt was associated with: Female gender (adjusted odds ratio (AOR) = 1.3; CI: 1.0, 1.7), exercise (AOR = 2.0; CI: 1.5, 2.5), and medications for COPD (AOR = 1.6; CI: 1.3, 2.2). Among black adults with COPD quit attempt was associated with: Having a partner (AOR = 4.5; CI: 1.3, 15.0), exercise (AOR = 3.7; CI: 1.6, 8.7), spirometry (AOR = 9.5; CI: 3.2, 28.7), and having a personal doctor (AOR = 6.4; CI: 1.8, 22.5). Individual and clinical-factors associated with quit attempt varied by race. These findings suggest an impact of the healthcare system that supports quit attempts in blacks but not whites with COPD.

## 1. Introduction

The majority of adult smokers make multiple attempts to quit before succeeding [1]. In adults with chronic obstructive pulmonary disease (COPD) only half report a smoking quit attempt in the past year even though tobacco smoking is the main risk factor for progression of COPD [2,3,4]. Smoking cessation in the COPD patient reduces respiratory symptoms, and is associated with improvement in health-related quality of life and increased survival [5,6,7,8]. Despite the benefits of smoking cessation, 36%–46% of adults with COPD are current smokers [4,9,10,11].

Quit attempts can vary by age and race/ethnicity. In the general population, older adults report lower interest in quitting when compared to younger adults and quit attempts decrease with age [12]. Research in the COPD population has shown that those more likely to make a quit attempt are younger [4]. Compared to white smokers, black smokers are more likely to report interest in quitting smoking and attempts to quit smoking are more common among black smokers [12,13]. Therefore, it is disappointing that blacks are less likely than whites to report cessation advice from a physician [14].

Guidelines for management of COPD include smoking cessation counseling; however, compliance to guidelines varies. In hospitalized COPD patients reporting current smoking, 30% to 69% were offered smoking cessation advice and 16% were offered nicotine replacement therapy [15,16]. In a national sample, 77% of adults with COPD reported that they were advised to quit smoking by a healthcare professional in the past year compared to 51% of adults without COPD [4]. A clinic-based study of adults with COPD found that 68% were provided with documented smoking cessation advice or treatment [17]. Reasons for the observed compliance to guidelines may be related to physicians’ negative beliefs and attitudes regarding discussing smoking cessation with their patients [18]. Although most physicians have positive beliefs and attitudes towards providing cessation advice, research has demonstrated that there is room for quality improvement in provision of cessation assistance in adults with COPD and smokers in general [18].

Ample opportunity exists in the clinical setting to assist adults with COPD in making a quit attempt. In the Medicare population, COPD ranks second highest in average yearly number of hospital discharges (X = 1.3) and inpatient days (X = 8.2) and it ranks third and fourth in physician office visits (X = 10.2) and institutional outpatient visits (X = 6.5) [19]. These encounters with the healthcare system are opportunities for health behavior intervention and may encourage quit attempts. This research examines the individual- and clinical-level factors associated with smoking quit attempts in adults with COPD as a step towards identifying opportunities for smoking cessation assistance.

## 2. Materials and Methods

### 2.1. Ethics Statement

This research involved the analyses of existing data and it was deemed minimal risk to human subjects and granted exempt status (did not require written informed consent) under federal regulation 45 CFR 46.101(b) by the Institutional Review Board at Kent State University (Protocol #13-306).

### 2.2. Study Population

Data for this cross-sectional study were derived from the 2011 Behavior Risk Factor Surveillance System (BRFSS) [20]. The BRFSS is a federally-funded telephone survey conducted annually by the Centers for Disease Control and Prevention in collaboration with the 50 state health departments and those in Washington, DC; Puerto Rico; the US Virgin Islands; and Guam. A representative sample of civilian, non-institutionalized adults 18 years or older was selected through a multistage cluster design and random digit dialing. The data from each state were weighted to reflect the respondent’s probability of selection and the age-by-sex or age-by-race/ethnicity-by-sex category in the population of the respondent’s state. To enhance sampling representation of the US population, the BRFSS used new methodology in 2011 that included the addition of cell-phone-only respondents. The Virgin Islands and Guam were excluded from the original release of data as census estimates were not available.

In 2011, respondents in all 50 states, Washington, DC, and Puerto Rico, were asked the question: “*Has a doctor, nurse, or health care professional ever told you that you have (COPD) chronic obstructive pulmonary disease, emphysema, or chronic bronchitis?*” An optional COPD module was then administered to respondents in 21 states if the respondent said “yes” that they had ever been told they had COPD, emphysema, or chronic bronchitis. These 21 states included: Arizona; California; Connecticut; Illinois; Iowa; Kansas; Kentucky; Maine; Massachusetts; Michigan; Minnesota; Montana; Nebraska; Nevada; New Jersey; North Carolina; Ohio; Oregon; Tennessee; Utah; West Virginia; and Washington, DC and Puerto Rico. The analyses for this study are limited to the responses from these states, Washington, DC, and Puerto Rico (*n* = 21,567).

Smoking history was identified through the following question: “Have you smoked at least 100 cigarettes in your entire life?” Of the *n =* 21,567 respondents, *n =* 16,504 responded “yes”, *n =* 5000 responded “no”, *n =* 58 responded “don’t know/not sure” and *n =* 5 refused to respond. To determine current or former smoking status, only the respondents who said “yes” were administered the following question: “*Do you now smoke cigarettes every day, some days or not at all*”. Of the *n =* 16,504 respondents, *n =* 5542 responded “every day”, *n =* 1596 responded “some days”, *n =* 9353 responded “not at all”, *n =* 8 responded “don’t know/not sure”, and *n =* 5 refused to respond. Respondents (*n =* 7138) who reported either “every day” or “some days” were retained for analyses.

### 2.3. Outcome of Interest: Attempt to Quit Smoking

Attempt to quit smoking (yes/no) was determined from the following question: “*During the past 12 months, have you stopped smoking for one day or longer because you were trying to quit smoking?*” Of the, *n =* 7138 respondents, *n =* 4452 responded “yes”, *n =* 2664 responded “no”, *n =* 18 responded “don’t know/not sure” and *n =* 4 refused to respond.

### 2.4. Covariates: Individual-Level and Clinical-Level

To examine factors associated with attempt to quit smoking among adults with COPD, the following covariates were included in analyses: Socio-demographic variables including age (continuous), marital status (partner *vs*. no partner (never married, divorced, separated or widowed)), gender, and education (high school graduate or GED or below, and some college/technical school or above); any exercise in past 30 days (yes/no); total medications taken for COPD (none, one or more); have a personal doctor (yes/no); seen a doctor about shortness of breath (yes/no); breathing test to diagnose COPD (yes/no); and been to the emergency room or hospital for shortness of breath (yes/no).

Respondents (*n =* 744) were excluded who responded “don’t know/not sure” or refused to respond on the outcome of interest or any of the covariates considered in this study or if they had any missing data. Respondents (*n =* 1049) were further excluded that were less than 40 years of age or if their ethnicity was Hispanic or multiracial non-Hispanic. This resulted in a final sample size of *n =* 5345 respondents with complete outcome and covariate data.

### 2.5. Statistical Analysis

Sampling weights that adjust for unequal selection probabilities, survey non-response, and oversampling were used to account for the complex sampling design. To describe the characteristics of the study population, weighted prevalence estimates, weighted mean estimates, and corresponding 95% confidence intervals (CI), were computed for the outcome of interest and all covariates considered. Univariate logistic regression was performed for each predictor with the outcome and variables maintained for multivariable regression if significant at *p* < 0.05. All predictors were retained in race stratified, multivariable logistic regression models in order to examine the effect of each factor within race on “attempt to quit smoking”. Analyses were conducted in SAS 9.2 (SAS Institute Inc., Cary, NC, USA) using survey procedures (PROC SURVEYMEANS, PROC SURVEYFREQ, AND PROC SURVEYLOGISTIC) to obtain correct variance estimates, using Taylor series linearization method.

## 3. Results

Overall, 65% (CI: 61.9, 67.5) of current smokers with COPD attempted to quit smoking within the past 12 months (Table 1). The sample was predominately female (61%) and white (91%). The mean age of respondents was 56 years (standard deviation *=* 0.3), and 63% were high school graduates or below. Fifty-seven percent of respondents did not have a partner (never married, divorced, separated or widowed). More than half (55%) of the sample reported having had any exercise in the past 30 days and 58% reported taking at least one medication each day for COPD. Healthcare utilization included: 77% who reported having had a breathing test to diagnosis their COPD, 90% who reported having a personal doctor, 40% who reported having seen a doctor for shortness of breath, and 15% who reported having been to the emergency room or been hospitalized for COPD in the past 12 months. Black compared to white adults were more likely to report a quit attempt (86% *vs*. 63%, respectively; *p* < 0.0001) and having had a breathing test to diagnose their COPD (88% *vs*. 76%, respectively; *p* < 0.01).

**Table 1:** Healthcare utilization factors and demographic characteristics of current smokers with COPD: frequency, weighted prevalence and 95% confidence intervals, overall and by race/ethnicity, 2011 BRFSS, *n* = 5345.

	Overall		White Non-Hispanic		Black Non-Hispanic		
	*n* = 5345		*n* = 4965		*n* = 380		
	*n* (%)	(95% CI)	*n* (%)	(95% CI)	*n* (%)	(95% CI)	*p*
**Attempt to quit smoking?** (Yes)	3299 (64.7)	(61.9–67.5)	3020 (62.7)	(59.7–65.7)	279 (85.7)	(80.0–91.4)	<0.0001
**Age**, mean (SD)	56.3 (0.30)	(55.8–56.9)	56.6 (0.32)	(56.0–57.2)	53.7 (1.03)	(51.7–55.8)	0.0097
**Gender**							0.0687
Male	1664 (39.4)	(36.3–42.6)	1556 (38.5)	(35.2–41.7)	108 (49.4)	(37.7–61.0)	
Female	3681 (60.6)	(57.4–63.7)	3409 (61.5)	(58.3–64.8)	272 (50.6)	(39.0–62.3)	
**Education level**							0.0233
High school graduate or below	3118 (62.7)	(59.8–65.5)	2915 (63.8)	(61.0–66.7)	203 (50.6)	(38.8–62.4)	
Some college, technical school or college graduate	2227 (37.3)	(34.5–40.2)	2050 (36.2)	(33.3–39.0)	177 (49.4)	(37.6–61.2)	
**Marital status**							0.0566
Never married/divorced/separated/widowed	3462 (57.0)	(53.9–60.0)	3138 (55.8)	(52.6–58.9)	324 (69.1)	(56.7–81.6)	
Married/member of unmarried couple	1883 (43.0)	(40.0–46.1)	1827 (44.2)	(41.1–47.4)	56 (30.9)	(18.4–43.3)	
**Exercise in past 30 days** (Yes)	2704 (54.7)	(51.7–57.7)	2501 (54.2)	(51.1–57.3)	203 (60.4)	(48.9–71.9)	0.3137
**Medications for COPD**							0.8319
None	2190 (41.6)	(38.6–44.6)	2043 (41.7)	(38.6–44.8)	147 (40.5)	(29.8–51.1)	
One or more	3155 (58.4)	(55.4–61.4)	2922 (58.3)	(55.2–61.4)	233 (59.5)	(48.9–70.2)	
**Alcohol consumption**							0.9789
Nondrinkers	3394 (62.0)	(59.1–65.0)	3172 (62.1)	(59.0–65.1)	222 (61.8)	(50.9–72.6)	
Light drinkers	1445 (27.7)	(25.1–30.3)	1308 (27.6)	(24.9–30.4)	137 (28.5)	(19.7–37.3)	
Moderate/heavy drinkers	506 (10.3)	(8.2–12.4)	485 (10.3)	(8.2–12.5)	21 (9.7)	(2.2–17.2)	
**Breathing test to diagnose COPD** (Yes)	4136 (77.2)	(74.5–79.8)	3831 (76.2)	(73.3–79.0)	305 (87.7)	(82.3–93.0)	0.0014
**Have a personal doctor?** (Yes)	4873 (89.8)	(87.7–91.9)	4519 (89.5)	(87.3–91.8)	354 (92.6)	(87.9–97.4)	0.2878
**Seen a doctor about shortness of breath?** (Yes)	2159 (40.4)	(37.4–43.4)	1981 (40.0)	(36.9–43.1)	178 (44.3)	(32.9–55.8)	0.4702
**Been to E.R./hospital for COPD?** (Yes)	765 (15.3)	(13.0–17.6)	677 (14.9)	(12.5–17.4)	88 (19.2)	(11.9–26.6)	0.2455

COPD = Chronic Obstructive Pulmonary Disease; BRFSS = Behavioral Risk Factor Surveillance System; CI = Confidence Interval; E.R. = Emergency Room; SD = Standard Deviation.

In race stratified, multivariable models, gender, exercise in past 30 days, medications for COPD, and alcohol consumption were the only significant factors for quit attempt in white adults (Table 2). When compared to white males with COPD, white females with COPD were 1.3 (CI: 1.0, 1.7) times more likely to have made a quit attempt in the past 12 months. Compared to white smokers who reported no exercise in past 30 days, white smokers who reported exercise were 2.0 (CI: 1.5, 2.5) times more likely to have made a quit attempt in the past 12 months. White adults who reported taking one or more medications each day for COPD compared no medications were 1.6 (CI: 1.2, 2.2) times more likely to have made a quit attempt in the past 12 months. Compared to white adult smokers who reported being moderate or heavy drinkers, white adult smokers who reported being light drinkers and nondrinkers were 1.7 (CI: 1.0, 2.8) and 2.1 (CI: 1.3, 3.4) times, respectively, more likely to have made a quit attempt in the past 12 months.

**Table 2:** Adjusted weighted prevalence odds ratios and 95% confidence intervals for making a quit attempt in the past 12 months among current smokers with COPD in the 2011 BRFSS, *n* = 5345.

	White Non-Hispanic	Black Non-Hispanic
	*n* = 4965	*n* = 380
	AOR (95% CI)	AOR (95% CI)
**Age**	1.00 (0.99–1.01)	1.00 * (0.96–1.05)
**Gender**		
Male	Reference	Reference
Female	1.32 (1.01–1.72)	1.83 (0.64–5.26)
**Education level**		
Some college, technical school or college graduate	Reference	Reference
High school graduate or less	1.07 (0.83–1.37)	2.49 (0.95–6.49)
**Marital status**		
Never married/divorced/separated/widowed	Reference	Reference
Married/member of unmarried couple	1.00 * (0.78–1.29)	4.49 (1.34–15.00)
**Exercise in past 30 days** (Yes)	1.96 (1.53–2.51)	3.71 (1.58–8.69)
**Medications for COPD**		
None	Reference	Reference
One or more	1.65 (1.25–2.16)	1.64 (0.53–5.07)
**Alcohol consumption**		
Moderate/heavy drinkers	Reference	Reference
Light drinkers	1.71 (1.05–2.77)	1.89 (0.45–8.02)
Nondrinkers	2.12 (1.33–3.36)	1.72 (0.33–8.88)
**Breathing test to diagnose COPD** (Yes)	1.12 (0.83–1.52)	9.53 (3.16–28.73)
**Personal doctor** (Yes)	0.88 (0.56–1.38)	6.43 (1.83–22.53)
**Seen a doctor about shortness of breath** (Yes)	0.96 (0.72–1.28)	0.64 (0.22–1.88)
**Been to E.R./hospital for COPD** (Yes)	1.43 (0.94–2.19)	0.64 (0.20–2.01)

COPD = Chronic Obstructive Pulmonary Disease; AOR = Adjusted Odds Ratio; CI = Confidence Interval; BRFSS = Behavioral Risk Factor Surveillance System; E.R. = Emergency Room; * OR = 1.002.

In black adults, the significant factors for a quit attempt in the past 12 months were having a partner, exercise in the past 30 days, having had a breathing test to diagnose their COPD, and having a personal doctor. Compared to black smokers who reported not having a partner, those with a partner were 4.5 (CI: 1.3, 15.0) times more likely to have made a quit attempt in the past 12 months. When compared to black smokers who reported no exercise in the past 30 days those who reported exercise in the past 30 days were 3.7 (CI: 1.6, 8.7) times more likely to have made a quit attempt in the past 12 months. Black adults who reported a breathing test to diagnose COPD compared to those who did not have a breathing test to diagnose COPD were 9.5 (CI: 3.2, 28.7) times more likely to have made a quit attempt in the past 12 months. Compared to black adults without a personal doctor, those with a personal doctor were 6.4 (CI: 1.8, 22.5) times more likely to have made a quit attempt in the past 12 months.

## 4. Discussion

Quit attempts in adults with COPD do not meet targeted recommendations for cessation attempts described by Healthy People 2020. In this national sample of current adult smokers with COPD, only 65% reported a quit attempt in the past 12 months. While this prevalence was lower than the Healthy People 2020 target of 80% by all current adult smokers it is higher than the 50% reported by Schiller and Ni [4,21]. Unfortunately, it is lower than expected in a population with debilitating chronic disease. Tobacco smoking is the main risk factor for the progression of COPD and it is estimated that 73% of COPD mortality is related to smoking in high-income countries [22]. Smoking cessation is the most important and cost effective method for delaying the progression of COPD and it is the only thing that reduces the decline in forced expiratory volume [2,5,23]; yet, 36%–46% of adults with COPD continue to smoke [4,10,24]. Identifying opportunities to facilitate quit attempts is an important step towards providing cessation assistance to adults with COPD.

Healthcare utilization factors associated with smoking quit attempts differed between black and white adults with COPD in the current research. In white adults with COPD, healthcare utilization factors were not related to quit attempts. This may be inherent to white adults or an oversight on the part of the healthcare system and deserves additional attention. Black adults with COPD who had spirometry to diagnose their COPD compared to those who did not have spirometry to diagnose COPD were more likely to report a quit attempt in the past 12 months. While a report by the Agency for Healthcare Research and Quality determined that the available evidence does not support that spirometry is effective in smoking cessation, the current research determined that it may play a role in quit attempts among black adults with COPD [8]. In addition, black adults who had a personal doctor compared to those who did not were more likely to report a quit attempt in the past 12 months. Interestingly, the direction of the relationship between having been to the ER or hospital and having seen a doctor about shortness of breath did not support a quit attempt; however, these emergency or inpatient services may be used more frequently by older or sicker people while the spirometry and personal doctor factors may be used by people who are seeking preventive care and aren’t quite as sick; perhaps these adults are trying to quit before their disease progresses and the ones using hospital/ER or seeing a doctor about shortness of breath are not willing to quit and have accepted their condition and smoking status. This idea could not be examined in the current study and warrants further investigation.

The current research was not able to determine if respondents were given advice to quit smoking; however, other research found that whites (48% *vs*. 33%) and blacks (50% *vs*. 38%) who received cessation advice by a physician had a higher prevalence of quit attempt than those who did not [25]. Also, research has shown that receipt of physician advice to quit smoking is significantly associated with quit attempt among whites (OR = 1.3, 95% CI = 1.2–1.4) and blacks (OR=1.5, 95% CI= 1.2–1.7) and that physician advice on smoking cessation is related to a 66% increase in the rate of quitting among smokers who received brief cessation advice and a slightly higher rate among those who received a more intensive intervention [25,26]. Regardless, encounters with the healthcare system provide unique opportunities to encourage quit attempts and smoking cessation with evidence that black adults with COPD are more willing to try to quit smoking in association with utilization factors.

In the current research, a greater proportion of black compared to white adults who were current smokers made a quit attempt in the past 12 months and this is consistent within the literature. For example, compared to white smokers, black smokers are more likely to report interest in quitting smoking and attempts to quit smoking are more prevalent among black smokers [12,13]. However, there are inconsistencies reported in success at quitting, with some reports observing better success in whites compared to blacks and some observing better success in blacks compared to whites [12,27]. In the general population, older adults report lower interest in quitting when compared to younger adults and quit attempts decrease with age [12]. Research in the COPD population has shown that those more likely to make a quit attempt were younger [4]; however, in the current research, there was no association with age in either race. In the current research, adults reporting any exercise in the past 30 days were more likely to make a quit attempt while previous research has observed that those more likely to make a quit attempt had activity limitation [4]; however, due to the methods describing exercise in the current research, those reporting any exercise may have been adults with limitations. Medications may be seen as a proxy for disease severity [2]. Therefore, white adults who were taking medications for COPD and were more likely to report a quit attempt may be trying to quit because they are sicker than those who were on no medications. That they were also more likely to report exercising in the past 30 days may be viewed as a preventative measure; however, this could not be examined in the current research.

This study has several potential limitations. First, cross-sectional data limits the assessment of the timing of the relationships between variables in the study and quit attempts. For instance, among black adults it is not known whether having a physician contributed to a quit attempt or if assistance for smoking cessation was needed and a physician was subsequent to a quit attempt. Second, not all relevant factors or potential confounders were assessed due to the limitations of the questions asked in the BRFSS. For instance, motivation or intention to quit is strongly associated with a quit attempt in the general population of smokers and its association with quit attempt should be considered in future studies among the COPD population [28]. Lastly, only white and black adults were included in the study due to sample size limiting the generalizability to other racial/ethnic groups. A major strength of this study was the utilization of a nationally representative sample of the United States population. Further, this nationally representative study is the first to our knowledge to examine utilization factors and their impact on quit attempt in adults with COPD of different races and observed differences in interactions with the healthcare system and quit attempts by race.

## 5. Conclusions

In summary, the available epidemiologic evidence indicates that quit attempts in the COPD population do not meet targeted recommendations delineated by Healthy People 2020. Previous research has documented that quit attempts vary with regard to race/ethnicity and age in the general population of smokers. The current results expand the body of literature by demonstrating the role the healthcare system plays in the likelihood of quit attempts among adults with COPD of different races. Clinical factors associated with quit attempt varied by race suggesting that there are opportunities for health behavior intervention in these populations that may support smoking cessation. Understanding race-specific factors that could result in successfully quitting in adults with COPD may increase cessation rates, reduce racial/ethnic disparities in tobacco behaviors and decrease the tobacco-related burden in this population. Future research will examine the impact of these individual and healthcare factors and their associations with cessation in adults with COPD of varying race/ethnicity.
